# LESSONS LEARNED FROM A USER-CENTERED DESIGN APPROACH RURAL OLDER ADULTS USING DIGITAL HEALTH TOOLS

**DOI:** 10.1093/geroni/igae098.2069

**Published:** 2024-12-31

**Authors:** Marquita Lewis-Thames, Shawn Trimble, Francesca Cejtin Rosen, Zachary Seigel, Kara Toll, Kensley Horne

**Affiliations:** Northwestern University Feinberg School of Medicine, Chicago, Illinois, United States; Northwestern University Feinberg School of Medicine, Chicago, Illinois, United States; Northwestern University Feinberg School of Medicine, Chicago, Illinois, United States; Northwestern University Feinberg School of Medicine, Chicago, Illinois, United States; Northwestern University Feinberg School of Medicine, Chicago, Illinois, United States; Northwestern University Feinberg School of Medicine, Chicago, Illinois, United States

## Abstract

Increasing age and rurality are associated with decreased care coordination. There is a growing interest in the role of digital tools in improving care across the care continuum for rural and older adult groups. Still, little is known about the best practices of rural older adults and digital health tool usability. Guided by a user-centered design approach, we 1) describe multiple strategies to meaningfully engage rural older adults in the development of a digital health tool to improve telehealth navigation and 2) report best practices assessed from our multi-modal iterative design approach. We engaged key informants, including rural older adults, their caregivers, providers, healthcare administrators, and an advisory board. Our mixed methods user-centered design approach triangulated findings through semi-structured interviews, a systematic literature review, tool usability testing, and a MyChart usability analysis using eye-tracking technology. Lessons learned from our analysis reveal that rural older adults readily engage with digital technology and often sustain use after their introduction. Digital health technologies were most commonly associated with relieving access to care and transportation barriers. Reduced internet access and challenges with using the equipment were common usability barriers. Useful strategies to overcome usability barriers include in-person or telephone tutorials and knowledgeable caregivers. Tools to assist rural older adults with technology barriers were limited. As digital health technologies increasingly integrate into rural care coordination, barriers faced by rural older adults must be considered. Research that aims to elevate the voices of rural older adults in digital health tool design is imperative.

